# Virtual Reality as a distraction therapy during cystoscopy: a clinical trial

**DOI:** 10.1590/0100-6991e-20223138-en

**Published:** 2022-05-05

**Authors:** DIEGO INÁCIO GOERGEN, DANIEL MELECCHI DE OLIVEIRA FREITAS

**Affiliations:** 1 - Hospital Nossa Senhora da Conceição, Urologia - Porto Alegre - RS - Brasil; 2 - Hospital Moinhos de Vento, Urologia - Porto Alegre - RS - Brasil

**Keywords:** Virtual Reality, Cystoscopy, Pain, Ambulatory Surgical Procedures, Realidade Virtual, Dor, Procedimentos Cirúrgicos Menores, Cistoscopia

## Abstract

**Objectives:**

to investigate whether virtual reality (VR) experience is associated with decreased pain sensation among patients who undergo rigid cystoscopy under local anesthesia.

**Methods:**

we performed a prospective, randomized, controlled study of 159 patients who were aleatorily enrolled into two groups: VR and control. VR experience intervention consisted of using a headset with a smartphone adapted to a virtual reality glasses where an app-video was played during the procedure. Main outcomes analyzed were pain, discomfort, heart rate variability, difficulty and duration of the cystoscopy. Statistical analyses were performed using a Student’s t test, Mann-Whitney test and Chi-square test. A P<0.05 was considered to be statistically significant.

**Results:**

among 159 patients studied (VR group=80 patients; control group=79 patients), the mean age was 63,6 years and 107 (67,3%) were male. There was no statistically significant difference in baseline characteristics between the 2 groups. VR was significantly associated to decreased heart rate variability (6,29 vs 11,09 bpm, P<0,001) and lower duration of the procedure (5,33 vs 8,65 min, P<0,001). Also, when cystoscopies due to double-J extraction were excluded, VR experience was associated with reduced pain on the visual analog score of pain (3,26 vs 4,33 cm, P=0,023).

**Conclusions:**

the use of VR as a distraction therapy while performing outpatient cystoscopies is safe, has no side effects, is associated with less pain and discomfort, and reduces length of procedure.

## RESUMO

**Objetivos:**

investigar se a experiência de realidade virtual (RV) está associada à diminuição da dor em pacientes submetidos à cistoscopia rígida sob anestesia local.

**Métodos:**

foi realizado um estudo prospectivo, randomizado e controlado de 159 pacientes que foram alocados aleatoriamente em dois grupos: RV e controle. A intervenção da experiência de RV consistiu no uso de óculos de realidade virtual com smartphone adaptado e fones de ouvido, onde um vídeo foi reproduzido durante o procedimento. Os principais desfechos analisados foram dor, desconforto, variabilidade da frequência cardíaca, dificuldade e duração da cistoscopia. As análises estatísticas foram realizadas com o teste t de Student, o teste de Mann-Whitney e o teste do qui-quadrado. Um P<0,05 foi considerado como estatisticamente significativo.

**Resultados:**

entre os 159 pacientes estudados (grupo RV=80; grupo controle=79), a média de idade foi 63,6 anos e 107 (67,3%) eram do sexo masculino. Não houve diferença estatisticamente significativa nas características basais entre os grupos. A RV foi significativamente associada à menor variabilidade da frequência cardíaca (6,29 vs 11,09 bpm, P<0,001) e menor duração do procedimento (5,33 vs 8,65 min, P<0,001). Além disso, quando cistoscopias devido à extração de duplo J foram excluídas, a RV foi associada à redução da dor na escala visual analógica (3,26 vs 4,33cm, P=0,023).

**Conclusões:**

o uso da RV como terapia de distração durante a realização de cistoscopias ambulatoriais é seguro, não tem efeitos colaterais, está associado a menos dor e desconforto e reduz a duração do procedimento.

## INTRODUCTION

Cystoscopy is an invasive diagnostic urological procedure used to evaluate hematuria, voiding disorders or irritative urinary symptoms. It consists of an insertion of a tube (flexible or rigid) equipped with a lens and light called cystoscope through the urethra that allows visualization of the lower urinary tract. Cystoscopy might be performed in an urology office or a hospital under sedation or local anesthesia. In the Brazilian public health system about 2500 cystoscopies are performed monthly, most of then on an outpatient basis and under local anesthesia^1^. However, some studies demonstrated that several patients still reports feeling pain during the procedure, although not at distressing levels and due to different causes^2^ ^,^ ^3^. Rigid cystoscopes and with larger sheets, male patients, first-time cystoscopies and cystoscopies with intent to investigate lower urinary tract symptoms were associated with more pain^2^ ^,^ ^3^.

Pain is a sensation that alerts the organism about potentially damaging stimuli. The human mind has a limited capacity and must be aware of pain in order to perceive it^4^. Therefore, it is possible to saturate the brain with various sensory and non-painful information in a way to reduce the perception of the painful stimulus^5^. In this scenario, cognitive distractions during medical procedures have been used in several contexts to reduce pain and, in some cases, to minimize the need for sedation^5^. The distraction therapy also has showed good results in alleviating pain in special populations, as pediatric oncology patients, burned victims, children undergoing procedures^6^ ^-^ ^8^.

Virtual Reality (VR) is a type of cognitive distraction that creates a visual and auditory immersion, lowering the noise and connection with the external environment^4^. VR systems consists of a hardware (headset, glasses, gloves, computers or mobile devices) and a software that provides a multi-context intervention environment^9^. It is an immersive and interactive experience, integrating multiple sensory levels and capturing a greater degree of attention^10^. 

Though VR is a technique still unknown by several medical doctors, it has been successfully used to minimize procedural pain in various situations as changing burn dressings, venipuncture, dental procedures, lipoma resections and minor surgeries^11^ ^-^ ^13^. 

Based on the hypothesis that the use of distractions might reduce discomfort and anxiety during medical procedures, we sought to analyze whether the use of VR during rigid cystoscopy is correlated to decreased pain when compared to patients not using this technology.

## MATERIAL AND METHODS

### Study sample

We performed a prospective randomized clinical trial that included patients who underwent elective rigid cystoscopy under local anesthesia between April 2019 to September 2019. Were excluded from the study patients younger than 18 years old, with cognitive or sensorial limitations (such as dementia or blind patients), and patients who refused to participate, or who underwent an aggregate procedure (such as urethral dilatation or meatotomy). The cystoscopies were subdivided according to their indications: diagnostic (hematuria; lower urinary tract symptoms); follow-up (after or during non-muscle-invasive bladder cancer therapy); or for double-J stent extraction.

The sample size was calculated based on an alpha error of 5% and a beta error of 20% (with a power of the study of 80%), with an average pain of 4,4 (on a scale from 0 to 10) and a standard deviation of 214. The study was designed to detect a 20% difference between groups. Thus, the calculated sample was 156 patients (78 in each group).

**Figure 1:**
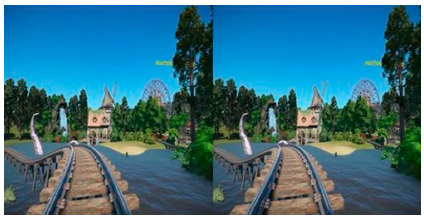
Screenshot of the app.

A Visual Analog Scale (VAS) was applied to each patient to assess pain and anxiety status (Figure 1). Difference between groups regarding VAS was analyzed in centimeters (cm).

Patients were randomized into two groups (VR experience and control). To avoid sample contamination, given that patients stayed in the same waiting room previous the procedures, randomization in blocks was performed (per day): every day, alternately, all scheduled patients were included in the control or the intervention group.

### Procedure technique

Cystoscopy with a rigid cystoscope was performed two minutes after intraurethral injection of 20ml of 2% lidocaine gel. The procedure was executed by one of a team of three medical residents in Urology. Decision to perform bladder biopsy was assessed during the procedure. As an objective parameter of pain, heart rate (HR) was monitored during cystoscopy and registered as variation over the basal HR on the peak. The duration of procedure was timed at the moment the cystoscope was introduced into the urethra and ended with its removal.

**Figure 2:**
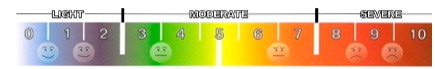
Visual analog scale.

VR experience: the patient was instructed about VR before the procedure at the time of consent. We used a Samsung^®^ A5 mobile phone adapted to Trust Urban^®^ Exos 3D virtual reality glasses and a headset. The patient placed the glasses on his face and positioned himself for the cystoscopy. On the screen, an app-video was playing simulating a ride on rails (screenshot at Figure 2) while the procedure was being performed. The chosen video had enough information to keep him distracted, but without sudden changes that could scare him and raise his heart rate. Despite the use of headphones, hearing communication was still maintained with the patient. After the procedure was done, the glasses were removed and the scales applied.

### Statistical Analysis

After the procedure, visual analog scales to evaluate pain and discomfort was applied (Figure 1). Side effects were also reported at this moment. After cystoscopy, the surgeon (a medical resident in Urology) described the difficulty level to perform the exam, on a scale of 0 to 10.

Main outcomes analyzed were: pain (on a VAS scale of 0 to 10), discomfort (on a VAS scale of 0 to 10), heart rate variation (on bpm), difficulty (on a VAS scale of 0 to 10), and duration (on minutes). Difference among scale rates were measured in centimeters (cm).

Baseline characteristics studied were gender (male/female), age (years), BMI (kg/m²), smoking (yes/no), ethnicity (white/black/others), number of previous cystoscopies, co-morbidities, cystoscope sheet (number 17/19/20), bladder biopsy (yes/no) (Table 1).

**Table 1:** Baseline characteristics.

Variables	VR (n=80)	Control (n=79)	p
Age, years (±SD)	62,11 (±13,35)	65,09 (±11,95)	0,141
BMI, kg/m² (±SD)	27,05 (±5,01)	26,72 (±4,86)	0,681
Pre-procedure Anxiety†, cm (±SD)	4,25 (±3,50)	4,43 (±3,54)	0,743
Pre-procedure HR, bpm (±SD)	76,26 (±11,49)	74,65 (±13,46)	0,455
Gender male, N (%)	57 (71,25%)	50 (63,3%)	0,285
Previous procedures			0,151
0	26 (32,5%)	22 (28,2%)	
1	13 (16,25%)	6 (7,7%)	
2+	41 (51,25%)	50 (64,1%)	
Ethnicity, n (%)			0,107
White	70 (88,61%)	61 (78,21%)	
Black	7 (13,92%)	9 (11,54%)	
Others	2 (2,53%)	8 (10,27%)	
Bladder Biopsy, n (%)	9 (11,25%)	13 (16,46%)	0,325
Type, n (%)			0,376
Diagnostics	17 (21,25%)	17 (21,52%)	
Follow-up	44 (55%)	50 (63,29%)	
Stent removal	19 (23,75%)	12 (15,18%)	
Cystoscope sheet, n (%)			0,679
Variables	VR (n=80)	Control (n=79)	p
17 Fr	13 (16,25%)	15 (18,99%)	
19 Fr	34 (42,50%)	36 (45,57%)	
20 Fr	33 (41,25%)	27 (34,18%)	

*p<0.05

†VAS scale (0-10)

Quantitative variables were analyzed using Student’s t-test for variables with normal distribution and Mann-Whitney for non-normal distribution. Categorical variables were analyzed using Chi-square test. The level of statistical significance was considered as p<0.05.

The study was approved by the IRB of Hospital Nossa Senhora da Conceição, under the CAAE number 08503119.9.0000.5530, and the clinical trial was registered on the Brazilian Clinical Trials Registry (ReBEC) under the Universal Trial Number (UTN) U1111-1235-8825. All patients consented for the study.

## RESULTS

A total of 175 cystoscopies were performed. Sixteen patients were excluded from the study due to: aggregate procedure (n=4), dementia (n=3), stent calcification (n=2), a blind patient (n=1), cystoscope malfunction (n=1), under age patients (n=2), presence of orthotopic bladder (n=1) and refuse of the patient (n=2). Among 159 patients enrolled, 80 patients were randomized to VR group and 79 patients to control group. There was no significant difference between the groups regarding age, BMI, pre-procedure anxiety, pre-procedure heart rate, gender, ethnicity, number of previous procedures, biopsy and type of procedure (Table 1). 

VR use was significantly associated to lower heart rate (p<0,001) and duration of the procedure (p<0,001) (Table 2). Surgeons reported lower difficulty during cystoscopies when patients were on VR experience when compared to controls (p=0,002) (Table 2). Subgroups analysis demonstrated that male patients were those who had more benefits on VR use (Table 3).

**Table 2:** Main outcomes.

	VR (n=80)	Control (n=79)	p
Pain†, cm	3,58 (± 2,31)	4,24 (± 2,88)	0,109
Discomfort†, cm	4,06 (± 3,01)	4,48 (± 3,06)	0,379
HR variation, bpm	6,29 (± 6,66)	11,09 (± 10,17)	<0,001*
Difficulty†, cm	1,66 (± 1,69)	2,81 (± 2,32)	0,002*
Duration, min	5,33 (± 3,17)	8,65 (± 5,01)	< 0,001*
Infecção	5	2.05%	
Hematoma	1	0.41%	
Problema na válvula	1	0.41%	

*p<0.05

*†VAS scale (0-10)*

**Table 3:** Outcomes subdivided by gender.

	Male			Female		
	VR (n=57)	Control (n=50)	p	VR (n=23)	Control (n=29)	p
Pain†, cm	3,63 (± 2,18)	4,46 (±2,84)	0,091	3,43 (± 2,64)	3,86 (± 2,96)	0,590
Discomfort†, cm	3,99 (± 2,97)	4,18 (± 2,80)	0,737	4,22 (± 3,18)	5,00 (± 3,45)	0,405
HR variation, bpm	6,46 (± 6,10)	12,09 (± 9,11)	< 0,001*	5,86 (± 8,03)	9,09 (± 11,99)	0,162
	Male			Female		
Difficulty†, cm	1,88 (± 1,79)	3,69 (± 2,25)	< 0,001*	1,13 (± 1,29)	1,34 (± 1,59)	0,923
Duration, min	5,32 (± 2,87)	9,46 (± 5,29)	< 0,001*	5,35 (± 3,90)	7,38 (± 4,32)	0,060

*p<0.05

†VAS scale (0-10)

When double-J extraction (n=31/19,5%) was removed from the sample, VR group described lower pain when compared to controls with significance (p=0.023) (Table 4). None of the patients related any side effect associated with the VR experience.

**Table 4:** Outcomes without the double-J removals.

	VR (n= 61)	Control (n=67)	p
Pain†, cm	3,26 (± 2,28)	4,33 (± 2,89)	0,023*
Discomfort†, cm	3,84 (± 2,96)	4,61 (± 3,08)	0,154
HR variation, bpm	6,53 (± 6,26)	11,25 (± 10,72)	0,005*
Difficulty†, cm	1,64 (± 1,53)	3,03 (± 2,36)	0,001*
Duration, min	5,72 (± 2,50)	9,28 (± 4,65)	<0,001*

*p<0.05

†VAS scale (0-10)

## DISCUSSION

Although several patients undergoing cystoscopy frequently complain about pain and discomfort during the procedure, there are several forms of distraction such as using a second screen for the patient, music, stress ball, or oral explanation to reduce pain^3^ ^,^ ^15^ ^-^ ^20^. We found that VR reduces heart rate variability, length of procedure and pain in patients who underwent cystoscopy.

The VR has been studied to alleviate pain and anxiety during diverse medical procedures. Hua et al. analyzed the use of VR as a non-pharmacologic intervention on a prospective randomized trial of 65 pediatric patients with chronic wounds on lower limbs who required active dressing changes. They demonstrated that patients who experienced VR showed significant lower pain on VAS scales and lower length of procedure^21^. Sharar et al. found similar results in burn victims throughout passive range of motion exercises, where patients reported not only diminished pain but also reduction on anxiety^22^. Controversially, Glennon et al. after analyzing pain scores in 97 patients on VR experience during bone marrow biopsy failed to indicate any difference between groups^23^. Nonetheless, controls also experienced another type of distraction other than VR, like music or television. Moreover, Wint et al. after investigating pain scores in 30 patients who underwent to lumbar puncture and were randomized to receive VR glasses versus standard of care, that included sedatives, found conflicting results. Of 30 patients who were enrolled, 17 were allocated on intervention group versus 13 on control group and no difference was found on pain visual analog scale between the groups^24^. However, in both studies, few patients were included and sample size was not calculated and different procedures were performed. Thus, despite the fact that VR use seems to be a promising intervention to alleviate pain, it is still a matter of debate and more studies with standardized designs and methodology are needed.

Some studies have reported the use of distraction techniques during cystoscopies to alleviate pain with mixed results. For example, Gupta et al. found that patients listening to music and with real-time visualization of the procedures during cystoscopies reported significantly lower pain when compared to controls. The authors also showed that in the intervention group post-procedural pulse rate was diminished in comparison to pre-cystoscopy (p<0.001)^17^. This is in line with our analysis, we found that patients who underwent to cystoscopies for causes other than double-J extraction had significantly lower increment in heart rate and diminished pain. In another study, Walker et al. randomized 45 patients before cystoscopy into two groups, with the VR immersion as intervention, failing in demonstrate any difference in pain improvement between the groups^14^. However, it was a small study and patients underwent to flexible cystoscopy, that is associated to lower pain than rigid cystoscopy^3^. Moreover, baseline characteristics that might influence in results, as gender and first-time procedure, were not assessed.

The relationship of non-pharmacological distraction therapies and pain reduction throughout medical procedures is an interesting question still under investigation. It has been acknowledged that pain perception is closely related to mind attention^10^. Melzack and Wall postulated that pain is perceived by the central nervous system as long as a painful stimulus travels through the body after passing certain nerve gates. This means that sensory pathways play a major role in pain awareness^25^. So, its plausible to think that VR immersion experience (that saturates several sensorial stimuli, like visual and auditory) may be an important tool to attenuate pain perception. Furthermore, pain sensation is also correlated to previous experiences and emotional status making painful threshold a very personal experience. 

The pain can be modulated by cognitive factors, like attention and distraction. Generally, the attention has to be actively directed to non-painful stimuli to avoid that painful stimuli prevail. Also, other painful stimulus can reduce de pain of previous painful stimuli^26^.

We found that VR during cystoscopy is associated with lower pain in subgroup analysis, reduced length of procedure, diminished heart rate variability and less difficulty to perform cystoscopy. The clinical implications of our findings are multiple. In our country most part of cystoscopies are outpatient basis procedures and VR implementation might be a routine because it is costless and with neglectable side effects. In addition, it might reduce the use of analgesics and narcotics during other medical interventions making it a not so an unpleasant experience. Furthermore, VR environment is a multiple distraction method and appears to be as effective as using multiple distraction approaches. Another important issue is that patient on VR seems to be less restless and more relaxed during the procedure, making it easier and faster for urologist to perform the cystoscopy. Consequently, these suggests that VR is an interesting intervention to be used in medicine.

Our study has several strengths, as a randomized controlled trial with a sizable sample. However, there are some limitations. First, mean age of our patients is over 60s and most of them are not familiarized with VR technology. Second, cystoscopies for double-J extraction were not excluded, and although this might influence in lowered length of procedure it also correlated to lower pain perception because it is faster than other cystoscopies as demonstrated on subgroup analysis. Third, there are other VR immersion technologies with different softwares and hardwares that might be compared in intervention group, because previous experiences occasionally may influence in painful threshold. Relaxing videos on VR immersive and interactive environment are probably more effective to attenuate pain than action videos. Fourth, all procedures were performed by medical residents in Urology who were in training and under learning curve.

Therefore, the use of VR as a distraction therapy was correlated with a reduction on the physiological response of pain (heart rate variability), as well as a lower distress during the cystoscopy, reducing the difficulty level and the time required to complete the procedure.

## CONCLUSION

Our study demonstrates that the use of VR as distraction therapy while performing outpatient cystoscopies is safe, has no side effects and reduces objective parameters of pain and discomfort, as well as facilitates the procedure and reduces its duration. The benefits of VR on reducing pain were significant in patients who underwent cystoscopy (excluding procedures for double-J extraction). However, given the fact that there are several new types of immersive technology of VR distraction, further studies are needed to analyze the feasibility of the method with more complex systems, as well as comparing it to other forms of distraction during the procedure.
